# Label-free, multi-scale imaging of *ex-vivo* mouse brain using spatial light interference microscopy

**DOI:** 10.1038/srep39667

**Published:** 2016-12-23

**Authors:** Eunjung Min, Mikhail E. Kandel, CheMyong J Ko, Gabriel Popescu, Woonggyu Jung, Catherine Best-Popescu

**Affiliations:** 1 Quantitative Light imaging Laboratory, Department of Electrical and Computer Engineering, Beckman Institute for Advanced Science and Technology, University of Illinois at Urbana-Champaign, 405 N. Matthews Avenue, Urbana, IL 61801, USA; 2 Translational Biophotonics Laboratory, Department of Biomedical Engineering, Ulsan National Institute of Science and Technology, 50 UNIST-gil, Ulsan 44919, Republic of Korea; 3 Department of Comparative Biosciences, College of Veterinary Medicine, University of Illinois at Urbana-Champaign, 2001 South Lincoln Avenue, Urbana, IL 61802, USA; 4 Center for Soft and Living Matter, Institute for Basic Science, 50 UNIST-gil, Ulsan 44919, Republic of Korea; 5 Cellular Neuroscience and Imaging Laboratory, Department of Bioengineering, University of Illinois at Urbana-Champaign, 208 North Wright Street, Urbana, IL 61801, USA

## Abstract

Brain connectivity spans over broad spatial scales, from nanometers to centimeters. In order to understand the brain at multi-scale, the neural network in wide-field has been visualized in detail by taking advantage of light microscopy. However, the process of staining or addition of fluorescent tags is commonly required, and the image contrast is insufficient for delineation of cytoarchitecture. To overcome this barrier, we use spatial light interference microscopy to investigate brain structure with high-resolution, sub-nanometer pathlength sensitivity without the use of exogenous contrast agents. Combining wide-field imaging and a mosaic algorithm developed in-house, we show the detailed architecture of cells and myelin, within coronal olfactory bulb and cortical sections, and from sagittal sections of the hippocampus and cerebellum. Our technique is well suited to identify laminar characteristics of fiber tract orientation within white matter, e.g. the corpus callosum. To further improve the macro-scale contrast of anatomical structures, and to better differentiate axons and dendrites from cell bodies, we mapped the tissue in terms of its scattering property. Based on our results, we anticipate that spatial light interference microscopy can potentially provide multiscale and multicontrast perspectives of gross and microscopic brain anatomy.

In order to better understand structure and function in healthy and disease states, the brain has commonly been imaged with whole-body imaging techniques such as Magnetic Resonance Imaging (MRI)^1^ and Computerized Tomography (CT)^2^. The main appeal of these techniques is that they can be applied to patients noninvasively. MRI, in particular, offers contrast of soft tissue and opportunities for functional investigation. However, both CT and MRI suffer from poor resolution, typically a few hundred microns. Even though the resolution can be enhanced up to a few tens of microns, it requires a great deal of time^3^,^4^. Optical methods, on the other hand, are capable of providing significantly better resolution and contrast. By taking advantage of this, the neural network was visualized at single neuron resolution by: optical coherence tomography (OCT)^5^, multi-photon fluorescence microscopy (MPM)^6^,^7^, Coherent anti-Stokes Raman scattering (CARS)^8^, stimulated Raman scattering (SRS)^9^, third harmonic generation (THG)^10^, and confocal reflectance microscopy^11^. However, most of these techniques still study brain tissue under a small field of view, thus have a limited ability to see the gross structure of brain. The other challenge in optical methods is the requirement of staining or the need for fluorescent tags, such as green fluorescence protein (GFP). The associated methods are time-consuming, costly, operator dependent, iterative, and often incomplete. In addition, co-staining is occasionally required to identify different parts of the neuron. For example, Luxol fast blue (LFB) stains axonal myelin, and Nissl dye stains the cell body (soma) of the neuron. Furthermore, in fluorescence imaging, the signal is unstable, and is degraded by photobleaching.

To address these obstacles, important progress has been made toward developing label-free, wide-field imaging methods: second harmonic generation (SHG)^12^, CARS^13^,^14^, THG^15^, Near-Infrared Reflectance (NIR) microscopy^16^, SRS^17^ and OCT^18^,^19^. These imaging modalities are predominantly sensitive to visualizing myeloarchitecture, but the image contrast is insufficient for delineation of cytoarchitecture. Specifically, SHG selectively detects non-centrosymmetric structures, e.g., microtubules inside the axons. The THG signal is obtained from the myelin sheath coating of axons in the phase-matching condition. SRS and CARS also detect the axonal myelin sheath via a strong signal from lipid CH_2_ stretch vibrations. In the case of CARS, non-resonant background noise occurs, which limits the signal to noise ratio. NIR has limited spatial resolution, because of its dependence on long wavelengths. Thus, the need for novel label-free imaging methods, of higher resolution and contrast, still exists.

Spatial Light Interference Microscopy (SLIM) is a quantitative imaging technique (QPI)^20^, capable of visualizing cytoarchitecture and myeloarchitecture without the use of exogenous labels in a non-contact manner. Conventional light microscopies such as Zernike phase contrast microscopy, and differential interference contrast (DIC) microscopy have been used to see the structure of neurons as well, but the images are *qualitative*, i.e., they do not render the pathlength map quantitatively. SLIM implements as a module attached to a Zernike phase contrast microscopy, extracts phase information in a *quantitative* way, with sub-nanometer sensitivity^21^. Thanks to these capabilities, SLIM has been used to study cell growth^22^, E. coli structure^23^, neural differentiation and network emergence^24^, cell tomography^25^, cancer diagnosis and prognosis^26^,^27^. However, with regard to brain imaging, so far the focus has been limited to imaging hippocampal neurons and glial cells in culture^21^. We performed, for the first time to our knowledge, SLIM in order to study the structure across the entire mouse brain. We can clearly recognize both the myeloarchitecture as well as the cytoarchitecture. In addition, we characterize the gross architecture of brain by analyzing the tissue scattering property, namely, the mean free path.

## Results

### Wide-field SLIM contrast

**Figure 1:**
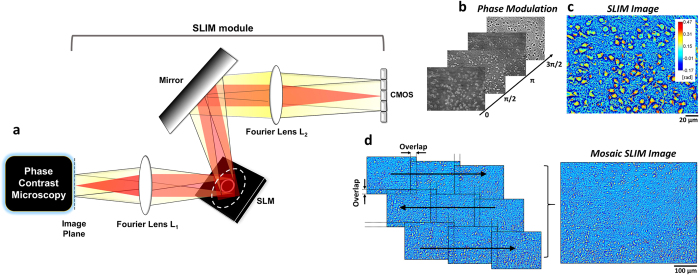
(**a**) The schematics of the spatial light interference microscope (SLIM). SLIM combines conventional phase contrast microscopy (left) and a SLIM module (right). The SLIM module consists of a 4f lens system, and spatial light modulator (SLM). The SLM is for phase modulation which is required for phase extraction. The yellow beam represents the unscattered light and the red beam presents the scattered light. (**b**) The four successive intensity images are taken when the phase of the unscattered beam is shifted by the SLM. The phase is shifted in increments of π/2 rad. (**c**) The SLIM image obtained by combining the four intensity images. (**d**) Wide-field SLIM image obtained by using an automatic wide-field acquisition and mosaic algorithm. There is 8% overlap between adjacent images.

**Figure 2:**
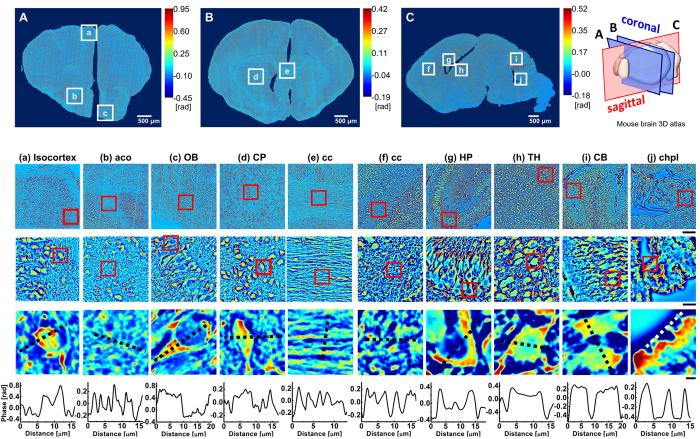
SLIM images of coronal (**A,B**) and sagittal (**C**) sections of the mouse brain at different spatial scales. The location of the brain being imaged is indicated by the white boxed inserts in A, B, and C. The location of the cross-section is depicted in the 3D atlas on the right. The red boxed insets include: isocortex (a), anterior commissure (aco,b), olfactory bulb (OB,c), Caudoputamen (CP,d), corpus callosum (cc, e and f), Hippocampus (HP, g), Thalamus (TH, h), Cerebellum (CB, i) and Choroid Plexus (chpl, j) at increasing levels of magnification. Row 1 scale bar = 100 μm, row 2 scale bar = 20 μm, and row 3 scale bar = 3 μm. The dotted black line drawn from left to right across each figure in row 3 is plotted to show the phase distribution and variation across the region imaged in row 4.

We imaged entire coronal and sagittal sections of the mouse brain with SLIM (Fig. 1). The images herein are displayed at different spatial scales. This makes it possible to obtain information about the morphological features of single neurons and myelin fibers, laminar structure, and distribution of nuclei and fiber tracts. In addition, we plotted the phase values of various regions of interest including the isocortex, anterior commissure (aco), olfactory bulb (OB), caudoputamen (CP), corpus callosum (cc), hippocampus (HP), thalamus (TH), cerebellum (CB), and choroid plexus (chpl) (Fig. 2). The phase values vary depending on the physical composition and contribution of cell bodies and axons/dendrites within a given region of interest.

SLIM provides morphological information from the phase shifts that occur when light passes through the brain tissue. The different phase values, between cell bodies and myelinated axons, for example, can be explained by relative differences in the phase delay caused by the relative thickness, density, or refractive index of the region of interest. The phase shift increases when tissue thickness increases, in areas of increased density and in areas where the refractive index is elevated. The axons and dendrites have lower phase values than the cell membrane and some organelles, e.g. the nucleus, because axon and dendrite diameters are relatively small. The areas typically known to contain cytoplasm and extracellular matrix are replaced by aqueous mounting medium during histological processing and consequently have the lowest phase values.

**Figure 3:**
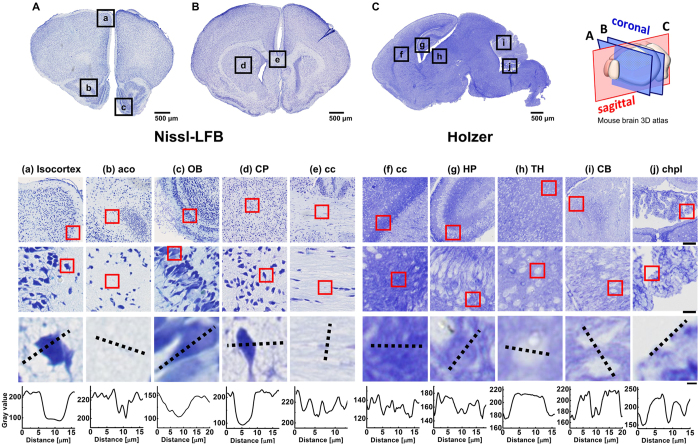
Histological coronal (**A,B**) and sagittal (**C**) section images of the mouse brain stained by Nissl-LFB (a-e), and Holzer (f-j) stains at different spatial scales. The location of the brain being imaged is indicated by the boxed inserts in A, B, and C. The location of the cross-section is depicted in the 3D atlas on the right. The red boxed insets include isocortex (a), anterior commissure (aco,b), olfactory bulb (OB,c), Caudoputamen (CP,d), corpus callosum (cc, e and f), Hippocampus (HP, g), Thalamus (TH, h), Cerebellum (CB, i) and Choroid Plexus (chpl, j) at increasing levels of magnification. Row 1 scale bar = 100 μm, row 2 scale bar = 20 μm, and row 3 scale bar = 3 μm. The dotted black line drawn from left to right across each figure in row 3 is plotted in row 4. Similar to SLIM contrast, the regions where cell bodies are densely packed have relatively lower Nissl-FLB and higher Holzer stain values in plot.

**Figure 4:**
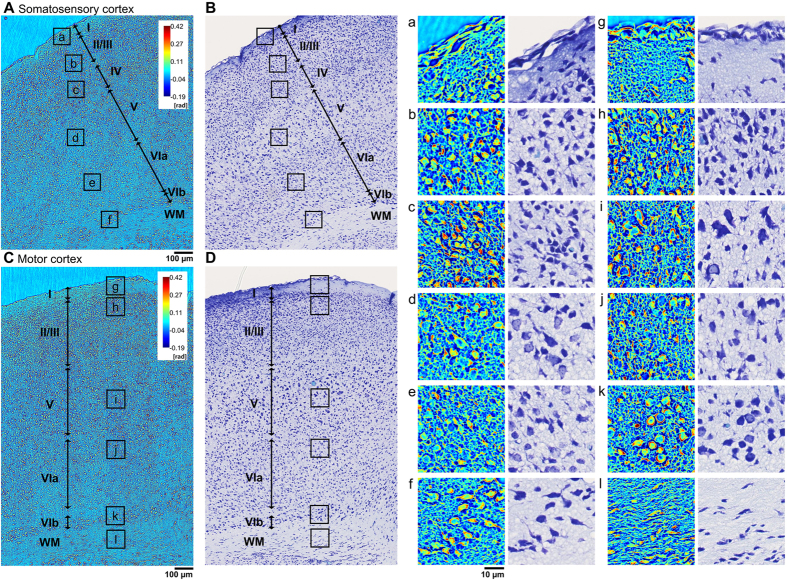
Laminar structure of isocortex of *ex-vivo* mouse, somatosensory cortex (**A** and **B**) and motor cortex (**C** and **D**) taken by SLIM (**A** and **C**) and stained with Nissl-LFB stain (**B** and **D**). Magnified images of layers I, II/III, IV, V, VIa and VIb: (a) molecular, (b) external granular and pyramidal, (c) internal granular, (d) internal pyramidal, (e), (f) multiform layer are also shown in the panels on the right. Magnified SLIM and Nissl stained images of Motor cortex boxed inserts (g), (h), (i), (j), (k) and (l) that correspond to Motor cortex layers I, II/III, V, VIa, VIb and WM in (**C**) are shown on the right. The layers are differentiated by the density and the distribution of the cells and by the types of cell present. WM, white matter.

**Figure 5:**
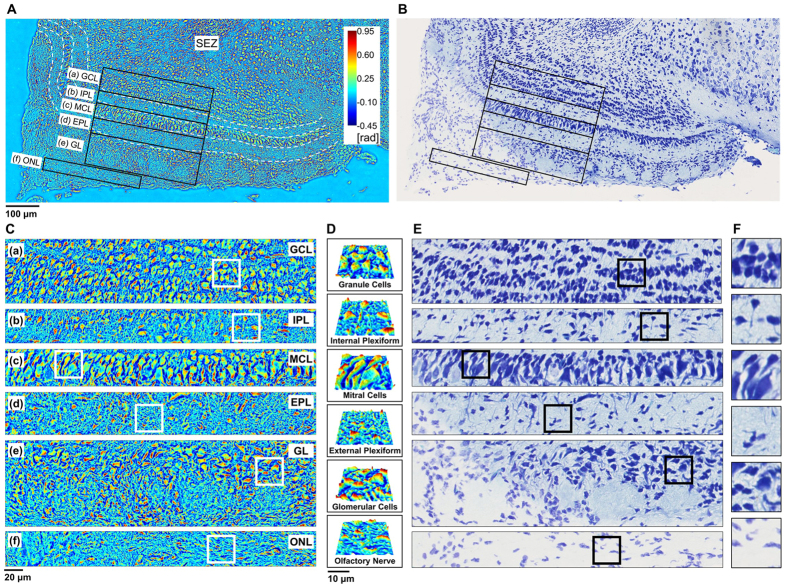
Anatomical structure of olfactory bulb (OB). (**A**) Laminar structure of OB taken by SLIM. (**B**) Nissl-LFB stained image corresponding to (**A**). (**C**) Enlarged SLIM images of OB layers which correspond to each layer outlined in (**A**). (**D**) Magnified topology of cells indicated by the white boxes in (**C**). (**E**) Enlarged Nissl-LFB stained images of OB layers which correspond to each layer outlined in (**B**). (**F**) Magnified topology of cells indicated by the black boxes in (**E**). GCL, granule cell layers; IPL, internal plexiform layer; MCL, Mitral cell layer; EPL, external plexiform layer; GL, glomerular layer; ONL, olfactory nerve layer, SEZ, subependymal zone.

**Figure 6:**
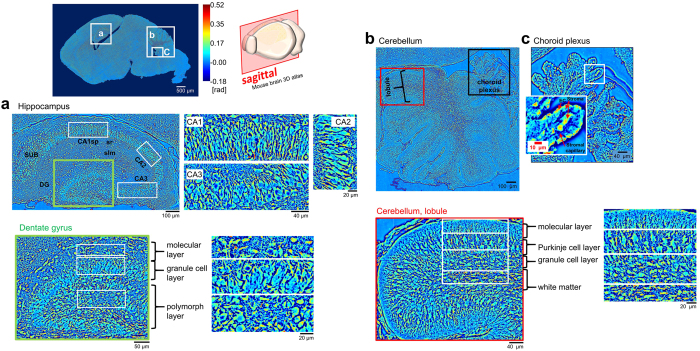
SLIM images of sagittal sections of the mouse brain. The location of the brain being imaged is indicated by the boxed inserts (**a**), (**b**) and (**c**), and the location of the cross-section is depicted in the mouse brain 3D atlas. Regions (**a**) hippocampus, (**b**) cerebellum and (**c**) choroid plexus are shown at increasing magnification. The green box in (**a**) shows the area of the dentate gyrus. The red box in (**b**) shows a cerebellum lobule. The layered structure for each region is analyzed in terms of phase value. The red arrows in (**c**) are pointing to stromal capillaries within the choroid plexus.

By mapping the phase values, the structure of brain tissue is well recognized, and comparable to the results obtained from standard histochemical analysis. For comparison, we stained coronal and sagittal mouse brain sections with Nissl-LFB and Holzer’s stain to differentiate cell body, myelin and glia, respectively (see Fig. 3). The laminar structure of the isocortex, OB, HP and CB are easily discerned by SLIM, respectively (see Figs 4, 5 and 6). The outer most layer of the isocortex, for example, consisting of sparsely spaced cell bodies and neuropil (demyelinated dendrites and axons) is easily separated from its contiguous adjacent layer that is comprised of densely packed neuronal cells (Figs 2a and 4). The aco, an anterior bundle of myelinated fibers that connects the two hemispheres at the midline of the brain (Fig. 2b) can clearly be distinguished from the subependymal zone (SEZ) of the OB. The SEZ is located next to aco, where the progenitor cells are closely adjoined. The six-layered structure of the OB is also easily differentiated (Figs 2c and 5). Likewise, the cc, the largest commissural fiber tract and the CP are adjacent structures that are clearly distinguishable by SLIM (Fig. 2d–f). This is because the cc consists of myelin fibers with a specific orientation angle that runs between the left and right hemispheres (Fig. 2e,f), and the CP includes a mixture of myelinated small-diameter axons interspersed with widely dispersed cell bodies (nuclei) (Fig. 2d). Unlike in the human brain, where the Caudate and Putamen are separated by an internal capsule, a white matter sheath, the CP is considered to be one anatomical structure in the mouse. The cc was imaged in the sagittal section as well for comparison and as expected, commissural fibers are shown in cross-section as they have been transected (Fig. 2f). Regions with densely packed nuclei corresponding to the HP, TH and CB are shown in Fig. 2g,h,i, respectively. Sagittal subfield regions of the HP, CB and chpl are (Fig. 2g,i,j) shown in greater detail (Fig. 6a,b,c). These SLIM images reveal the characteristically arranged jelly roll pattern of the HP, the folia of the CB, and the border of the ependymal cells with secretory structures typical of the chpl. The above structures will be described in more detail in following section. The gross structure of the brain was charted with SLIM, and to improve the macro-scale contrast of anatomical structures, and to better differentiate axons and dendrites from cell bodies, we plotted the tissue in terms of its scattering property.

### Qualitative comparison between Nissl-LFB stain and SLIM contrast

We compared the SLIM images to their corresponding Nissl-FLB stained coronal histological sections (Fig. 3a–e). The SLIM images are well matched with the Nissl-FLB stained images. The cell bodies having high phase values in the SLIM images correspond to the cells stained with Nissl dye. Similarly, the axon and dendrite having low phase values corresponds to myelin stained with LFB dye. The results clearly show that the SLIM images are not only qualitatively comparable to the Nissl-FLB stain image, but that they have improved contrast. The membrane, axon and organelles are clearly differentiated, in addition SLIM provides quantifiable information about cellular topography.

The tissue, in sagittal section, was also stained with Holzer to identify glial fibers (Fig. 3f–j). However, glial cells were not recognized with our SLIM (Carl Zeiss, EC Plan-Neofluar 40 × /0.75 Ph2), but the cell bodies of neurons are seen since the numerous glial cells surround and support neurons.

### Anatomical structure imaging based on SLIM contrast

Cellular attributes shown in the SLIM images including the cytoarchitecture, myeloarchitecture and topologies of regions around the isocortex, OB, cc, HP, CB and chpl are presented in detail in this section. We explain the structure of the mouse brain with the knowledge of neuroanatomy.

### Isocortex

The isocortex is a band of gray matter that covers the cerebral hemispheres and coats the outside of brain as shown in Fig. 4. In contrast to human, and other higher animals, the isocortex of mouse is lissencephalic (it is smooth and lacks gyri and sulci). The phylogenetically most recent part of the isocortex, the neocortex, has six distinct cell layers (I-VI) that vary in cell type and morphology, cell density, and layer thickness. The somatosensory (Fig. 4A and B) and motor cortex (Fig. 4C and D) were analyzed, and our analysis delineates six sublayers (I, II/III, IV, V, VIa, VIb). The unique cytoarchitecture and myeloarchitecture of each layer is shown in detail (Fig. 4a_l). Layer I, the molecular layer, is easy to distinguish from the other layers, because it is comprised primarily of dendrites and axons, (few neuron cell bodies) and consequently has the lowest phase values. Layers II and III are the external granular and external pyramidal layer, respectively. In the mouse, layers II and III are not distinguishable and are referred to as layer II/III. It contains densely packed small interneuron cells, granule (stellate) cells and numerous cone shaped pyramidal neurons. The boundary between the layer I and II/III is clearly seen. Layer IV, the internal granular layer, is composed of granule cells, which exists in the somatosensory cortex as a thin layer but is absent in the motor cortex. Layer V, the internal pyramidal layer is easy to discern, because it contains large pyramidal neurons. Layer VI, the multiform layer, is subdivided into VIa and VIb. Layer VIa contains the fewest number of cells, and VIb contains the elongated spindle-shaped fusiform neurons.

### Olfactory Bulb (OB)

The olfactory bulb (OB) contains the first synaptic relay in the olfactory pathway. It has a characteristic six-layered laminar structure containing distinct populations of neurons^28^. The SLIM image of laminar structure is clearly depicted (Fig. 5A). The olfactory nerve layer (ONL), glomerular layer (GL), external plexiform layer (EPL), Mitral cell layer (MCL), internal plexiform layer (IPL), granule cell layers (GCL) and SEZ region are all clearly delineated. The mitral cells have the largest cell bodies and nuclei in the OB, and they form a thin band- the MCL. Mitral cell axons serve as the major outflow of the OB. The axons of the olfactory sensory neurons (OSNs) form the outermost layer of the bulb and together they form the ONL- the input area of the OB. They carry olfactory information to, and form synapses with, second-order projection neurons (glomeruli) in the GL. Apical dendrites of the mitral cells innervate the glomerulus, and lateral dendrites of the mitral cells synapse extensively with another population of interneurons- the Granule cells. The glomeruli are circle shaped, and are surrounded by interneuron cell bodies, and fiber-like structures that are comprised of a combination of axons from the OSNs and dendrites from the Mitral cells^29^. The cellular layers, MCL and GL, and GCL and MCL, are separated by a layer of axons and dendrites, the EPL and IPL, respectively. Characteristic Mitral, Granule cell and Glomerular morphologies are compared in Fig. 5D. The laminar structure of OB is also seen in the Nissl-LFB stain image (Fig. 5B) and it is enlarged in Fig. 5E and F.

### Hippocampus

The hippocampus is a curved medial temporal lobe structure with rostrocaudal topographic orientation. In mice, the hippocampus is more dorsally oriented than in humans, and is arranged in two U-shaped interlocking regions, the dentate gyrus (DG) and the hippocampus proper, the Ammon’s horn (Cornu Ammonis (CA) subfields CA1-CA3) (Fig. 6a). The hippocampus is further characterized by multiple cell types with extensive afferent, efferent and intrahippocampal connectivity. The CA subfields, CA1, CA3 are predominately comprised of triangular pyramidal neurons with dendritic spine, in which they have higher value of phase than the regions of stratum radiatum (sr) and stratum lacunosum moleculare (slm). The hippocampal CA2 region is very narrow compared to CA1 and CA3, and typically is hard to detect without fluorescence labeling. The DG is a densely cellular band populated by granule neurons. It is comprised of three internal layers (see the green box in Fig. 6a): (1) The molecular layer (ML), which is comprised of the granular cell apical dendrites and their afferents; (2) The granule cell layer (GCL) which is made up of the densely packed granule cell bodies; and (3) the polymorph layer (PL) is comprised of interneurons, basket cells. The granule cell axons merge together to form the mossy fiber bundle and extends to the CA3 field.

### Cerebellum

The cerebellum consists of two cerebellar hemispheres joined by a median vermis. It is partitioned into lobules, and each lobule is subdivided into tightly folded layers (folia), separated by numerous parallel transverse folds and fissures. A sagittal section through the cerebellum, made parallel with the median plane, results in the distinctive branched appearance called the arbor vitae (tree of life) (Fig. 6b). The cerebellum contains 60% of all brain neurons in the mouse^30^, and like the isocortex and the OB, is composed of a highly organized laminar structure. The cerebellum is made up of a four cortical layers (see the red box in Fig. 6b): the external molecular layer (ML), the middle Purkinje layer (PL), and the internal Granular layer (GL), and a central white matter (WM) core. The ML layer has lowest phase value among other layers because it contains two types of inhibitory interneurons, the outer stellate and inner basket cells embedded among vast Purkinje cell dendritic arbors. Purkinje dendrites, studded with hundreds of short thick dendritic spines make up extensive flat dendritic arbors that extend into the ML where they branch profusely. These arbors lie in parallel planes with neighboring Purkinje arbors. In addition, the granule and Golgi cells thin axons run parallel to the long axis of the folia toward the ML. The PL is made up of inhibitory integrative Purkinje cells (PCs) arranged in a single layer, Candelabrum interneurons and Bergmann glia^31^. The large flask-shaped PCs are easily discernable among other cells in the border between ML and GL. In the SLIM image of the PCs (Fig. 6b), they are surround by fibers; the PCs receive excitatory input from 100,000–200,000 parallel fibers, and input from the contralateral inferior olivary nucleus via a single climbing fiber. Purkinje axons make up the primary efferent pathway from the cerebellar cortex. The axons arise from the base, pass through the GL, acquire a myelin sheath and enter the WM. Multiple collateral axon branches synapse with basket and stellate cells of the ML. The GL, is a densely packed layer containing small granule cells, slightly larger unipolar brush cells, Lugaro cells, larger Golgi cells and mossy fibers. Each granule cell has four or five dendrites, and an axon that bifurcates in the ML making distinctive T junctions with branches running parallel to the long axis of the folium. These fibers run perpendicular to the Purkinje cell dendrites.

### Choroid Plexus (chpl)

The chpl is a vascularized, villous secretory structure that arises from the walls of all four ventricles. Chpl cells are modified ependymal cells. In addition to having special transport and barrier functions, the chpl produces cerebrospinal fluid. The tissue contains multiple branching fronds that extend into the ventricles. The cells form a single layer of cuboidal epithelial cells on the apical side, and overlay thin walled capillary loops bound by a thin band of collagenous connective tissue (the stroma) (Fig. 6c). The epithelial cells rest on the basal lamina, and have long bulbous microvilli projecting from the luminal surface.

### Corpus Callosum (cc)

**Figure 7:**
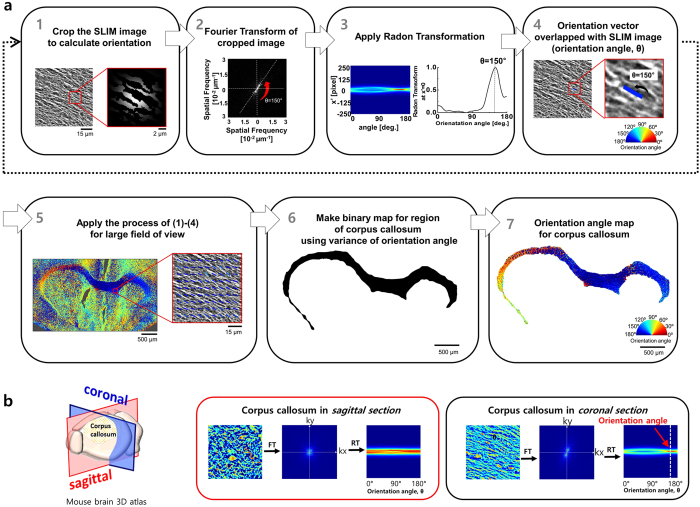
(**a**) Flow chart for orientation extraction from the corpus callosum (cc). See text for more details. (**b**) Comparison of the cc fiber orientation angles in coronal and sagittal sections. To estimate the 2D orientation angle, we applied Fourier transform (FT) and Radon transform (RT), and the orientation angle is estimated from RT generated intensity profiles.

The cc is the largest white matter tract that connects the left and right hemispheres. It contains approximately 7 million fibers in the mouse. The ability to quantify myelin amount (or volume), and the quantification of changes in myelin structure, is important to researchers studying demyelinating diseases such as Multiple Sclerosis^32^. In our analysis, we focus on fiber orientation which may potentially serve as a critical biological marker for studying Central Nervous System (CNS) diseases such as Traumatic Brain Injury (TBI). Figure 7 shows a flow diagram that describes the process of orientation angle extraction^33^.

**Figure 8:**
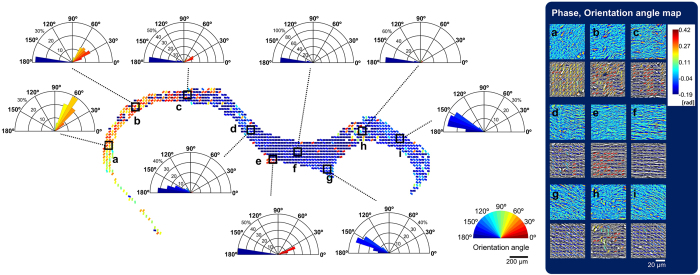
Myeloarchitecture of corpus callosum (cc). The orientation angle of the myelin fibers was calculated for several regions (**a–i**) and analyzed by orientation angle histograms in polar coordinate plots. In the histograms, the frequency distribution, as a percent of the fibers, with respect to the orientation angle are displayed. In the middle of the cc, most of fibers (80%) are aligned in parallel (**f**) and almost no cell bodies are shown, whereas, some cell bodies are distributed in other region of cc. The alignment of the fiber becomes irregular as it nears the cingulum bundle (**c,h**) and at the fiber terminal (**a,b,i**).

In particular, the fibers in coronal section, were analyzed, where they are aligned in parallel. In this process, we first cropped the SLIM image for small region of interest. The window size for crop was 15 × 15 μm^2^ (1 in Fig. 7a). Thus, the minimum readable spatial frequency is about 5/mm according to the uncertainly relation. Then, Fourier transform (FT) was applied on the cropped image after a windowing Gaussian function (2 in Fig. 7a). A Radon transform (RT) was then performed in the spatial frequency domain (3 in Fig. 7a). We extracted the orientation angle from the x′ = 0 graph and the result is overlapped with the SLIM image (4 in Fig. 7a). By calculating all the regions of brain with this manner, an orientation angle map was successfully created (5 in Fig. 7a). We built a binary mask for cc part using the fact that the orientation value in the fiber tract is continuous (6 in Fig. 7a), whereas, orientation angle in other regions is irregular. The binary mask was multiplied by the orientation map, and thus the orientation map only for cc was obtained (7 in Fig. 7a). Additionally, the orientation of fibers in the sagittal section were analyzed, however there was no specific orientation value since the cross-section is perpendicular to the fibers (Fig. 7b). The orientation angle changed across the entire cc is presented in polar coordinate (Fig. 8). In most regions, the orientation in horizontal direction is predominant. Particularity, in the middle of the cc, the fibers strictly align in a horizontal direction. Close to the cingulum bundle, and at the terminal of the cc fiber tract, the direction of the fibers are much more variable.

### Anatomical level contrast using inherent optical property

**Figure 9:**
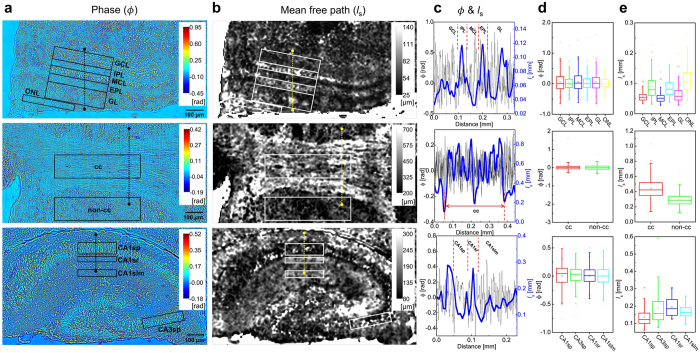
Comparison between SLIM and mean free path for the regions of the olfactory bulb (OB, row 1), corpus callosum (cc, row 2) and hippocampus (HP, row 3). (**a**) The SLIM image and the corresponding (**b**) mean free path map. Mean free path maps (**b**) of the OB, cc and HP laminar structure match the anatomical structure shown in the corresponding SLIM images (**a**), but with macroscopic contrast. (**c**) The phase and mean free path for the area marked by the dotted line drawn from top to bottom are analyzed and plotted. (**d**,**e**) Comparison between the phase and mean free path for the regions indicated by the black and white boxes in (**a**) and (**b**), respectively using box plots. The anatomical boundaries of tissue are more clearly identifiable by the mean free path (**e**) than phase value (**d**).

SLIM quantifies subcellular structure at the micro-scale, however, the contrast was not sufficient to recognize large-scale properties at the anatomical level. So, we used mean free path analysis which enhanced the contrast, similar to MRI or OCT. This is because the brain is composed of cell bodies and axons and dendrites. The varying geometrical shapes and spatial inhomogeneity of these structures throughout the brain results in different corresponding refractive indices and differential light scattering effects. Thus, the varied scattering properties between cell bodies, and axons and dendrites, gives us a new intrinsic contrast property to visualize gross structure of brain. Scattering of the light was quantified in terms of mean free path (the reciprocal to scattering coefficient). In general, intensity with respect to depth is experimentally obtained to calculate mean free path according to Beer’s law. However, the scattering-phase theorem we used here only required the 2D phase image to calculate the mean free path. We applied the scattering-phase theorem^34^ to the entire *en-face* brain slice, particularly regions of OB, cc and HP part were analyzed. The window size for mean free path calculation was 12 × 12 μm^2^. The mean free path for the regions was compared with their respective SLIM images (Fig. 9a and b). The phase and mean free path for the area marked by dotted line and boxes were analyzed and compared (Fig. 9c–e). From this result, we found that the phase for the different regions was similar. The mean free path, on the other hand, revealed significant differences depending upon the physical composition of the tissue. The highest mean free path values correspond with the EPL and IPL, and lowest mean free path values correspond with the GL, MCL and GCL in the OB (Fig. 9 first row). The cc (Fig. 9 second row) has a lower mean free path than its surrounding area. In the HP (Fig. 9 third row), the CA1 sp, sr, and slm subfields have lower phase values compared with the pyramidal cell layers. The entire section of the brain was compared in this manner with SLIM. SLIM image analysis bridges the gap between micro/nano-scale and macro-scale imaging. This approach opens new possibilities in the field of neuropathobiology given that we can now analyze changes across multiple-scales.

## Discussion

Here we highlight the use of SLIM, a phase contrast based microscopy technique, to generate high resolution images of *ex-vivo* mouse brain coronal and sagittal cross-sections. In previous papers, only single neurons in culture were imaged with SLIM. We demonstrate wide-field image analysis for entire *en-face* sections for the first time. One of the most significant advantages in brain imaging is measuring nanoscale neuron cell structures without exogenous contrast agents. We demonstrate unprecedented quantitative cytostructural information across multiple regions in the brain at the anatomical level. Another advantage of using SLIM is that we are not limited by non-uniform staining, or limited to rodent only models, e.g., green fluorescent protein is currently limited only to rodent models.

Based on our results, we found that neuronal cell bodies have higher phase values than myelinated/demyelinated axons, and dendrites. The cell membrane, axon and dendrites have much higher phase values than cytoplasmic regions, and the extracellular matrix. We show the laminar patterns of the isocortex, the OB, the HP and CB using phase contrast. Furthermore, the acquired SLIM images are directly comparable to the current gold standard Nissl-LFB histological stained sections (Figs 4 and 5). Overall, the cells and lamellar structures showed comparable features, and the SLIM image results matched well with the Nissl-LFB stain images. The differentiating contrast is better in SLIM. Therefore, with our new approach, we are able to map neuronal structure within brain tissue with unprecedented detail. Neuronal cytoarchitecture in the small field of view is well visualized by assessing phase contrast, but variations at the anatomical level are hard to quantify by phase analysis alone. Thus, in order to enhance macro-scale contrast, we assessed a scattering property, i.e., mean free path. As a result, quantifiable visualization of anatomical structure in the OB, cc and HP subregions of brain, was achieved (Fig. 9). Furthermore, we introduce a new method to quantify and characterize white matter fiber tracts. We utilized digital signal processing to find the orientation angle of myelinated axons in the largest commissure- the cc. We found that the phase values corresponding to myelinated fibers in the fiber tract are similar to the observed phase values of randomly distributed myelinated axons and dendrites. Thus, to distinguish between these, we adopted a RT algorithm and extracted the orientation angle of the fiber tract from the RT generated intensity profiles (Fig. 7). And we mapped the orientation angle of myelin fibers throughout the cc (Fig. 8).

In the future, we hope to better differentiate neuronal subtypes from glia, as well as to be able to distinguish axons from dendrites. These results indicate that, in principle, SLIM can image an entire mouse brain sliced in thin layers and computationally render it in 3D. This reconstruction will allow us to observe the complete brain connectome across the nanometer to the centimeter scale. This is a proof of concept, we show that SLIM is able to differentiate white matter tracts across both sagittal and coronal sections, and that we are able to resolve areas of white matter (myelinated axons) with SLIM and mean free path analysis. Thus, our next step is to reconstruct 3D neural network maps using SLIM. While dealing with such an enormous amount of data is challenging, this task has been demonstrated using chemical staining^35^,^36^ and fluorescence imaging^37^. In contrast to the fluorescence imaging of the whole brain, SLIM will provide a detailed view of the entire dry mass distribution of the brain, not just the structures that are tagged with a given fluorophore. This novel type of information is clearly complementary to that retrieved via fluorescence. In addition, SLIM can readily be coupled with fluorescence to incorporate molecular and cellular specificities, and to help with characterizing the distribution of specific molecules across the entire brain. Furthermore, SLIM is complimentary to a number of currently used techniques, e.g., organotypic cell culture that requires real-time imaging, with the added benefit of no loss of fluorescence, seen in photobleaching, for long-term monitoring at single cell resolution scales.

## Method

### Brain Tissue Specimens

All animal procedures were carried out in tight accordance with the recommendations in the Guide for the Care and Use of Laboratory Animals of the National Institutes of Health. Our animal protocol was approved by the University of Illinois Animal Care and Use Committee (IACUC) USDA registration: # 33-R-0029, NIH Animal Assurance PHS: # A3118-01 (Protocol Number: 14222). Brains from C57BL mice were processed according to standard procedures. Specifically, whole brains were fixed in 4% paraformaldehyde for 24 hours, at 4 °C, dehydrated and embedded in paraffin, and 4 μm sectioned. Each brain slice was then deparaffinized with xylene, rehydrated, and mounted in aqueous mounting media to recover the refractive index profile in tissue. SLIM images were subsequently obtained from the deparaffinized tissue specimens. After SLIM analysis, the coronal samples were stained with Nissl-LFB dye according to standard Kluver and Barrera procedures and the sagittal sections were stained with Holzer’s stain. The corresponding sections were imaged with a NanoZoomer Digital Pathology slide scanning system (Hamamatsu) for comparison.

### Spatial Light Interference Microscopy (SLIM)

SLIM is comprised of a commercial phase contrast microscope (Carl Zeiss, Axio Observer.Z1) combined with a SLIM module (Phi Optics, Inc., Cell Vista SLIM Pro) as depicted in Fig. 1a. The system uses white light from a halogen lamp, with a center wavelength of 550 nm. Due to the white light spectrum, SLIM provides inherent optical sectioning, with 1.2 μm temporal coherence length. The scattered and unscattered light fields emerging from the brain sample are further discriminated in the focal plane of the objective (Carl Zeiss, EC Plan-Neofluar 40 × /0.75 Ph2). The transverse resolution is approximately 0.4 μm. The objective focal plane is relayed by the SLIM module onto a spatial light modulator (SLM) (SLM; Boulder Nonlinear Systems, HSP512-635), which allows us to control the phase delay between the scattered and unscattered light. The SLIM module also contains a 4f system, which relays the output image at the camera (CMOS; Andor, Zyla 4.2). Interference occurs when the optical path length between the reference and sample light are matched within the temporal coherence length. The interference signal on the sample plane at position r = (x,y) is expressed as:


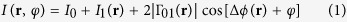


*I*_0_ and *I*_1_ are intensity of reference and sample respectively, and Γ_01_ (r) is the temporal cross-correlation function between the two fields evaluated at the origin. *φ* presents the phase shift between the scattered and unscattered fields. To retrieve phase information, a 4-step phase-shift interferometry technique was applied. The four SLIM images correspond to reference beam phase shifts of 0, π/2, π, and 3π/2 (Fig. 1b). The phase modulation process, and result, are shown in Fig. 1c. The phase delay, Δ*Φ* (r) is calculated by^25^:


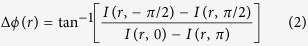


And, the phase map associated with to the image field is:


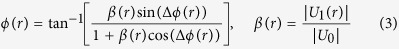


Where *U*_0_ and *U*_1_ are, respectively the unscattered and scattered light. The spatial sensitivity of phase in terms of optical path length change was measured as 0.3 nm, which is largely due to the absence of speckles associated with the white light illumination. For wide-field imaging, the XY scanning stage (Märzhaüser Wetzlar, SCAN IM 130 × 100) was integrated with the sample stage and we imaged entire coronal cross-sections of an *ex-vivo* mouse brain. The field of view of a single image is 156 × 208.8 μm^2^ (1040 × 1392 pixels). All SLIM images were stitched by using home-built software and FIJI, with overlapping set at approximately 8% (Fig. 1d).

To have macroscopic contrast imaging of the brain, the scattering property, i.e., mean free path (*l*s) was calculated from SLIM images using the phase-scattering theorem, which is described as:


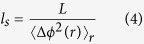


Where <∆*ϕ*^*2*^ (r)>_r_ and *L* indicate the phase variance, and thickness of brain specimen, respectively. In the above equation, mean free path is inversely proportional to phase variance. This means that phase fluctuations are related to how much scattering occurs in tissue.

## Additional Information

**How to cite this article**: Min, E. *et al*. Label-free, multi-scale imaging of *ex-vivo* mouse brain using spatial light interference microscopy. *Sci. Rep.* **6**, 39667; doi: 10.1038/srep39667 (2016).

**Publisher's note:** Springer Nature remains neutral with regard to jurisdictional claims in published maps and institutional affiliations.
